# On-Line Measurement of Tracking Poses of Heliostats in Concentrated Solar Power Plants

**DOI:** 10.3390/s24196373

**Published:** 2024-10-01

**Authors:** Fen Xu, Changhao Li, Feihu Sun

**Affiliations:** 1 School of Electrical & Control Engineering, North China University of Technology, Beijing 100144, China; lich329@chinaunicom.cn; 2 Institute of Electrical Engineering, Chinese Academy of Sciences, Beijing 100190, China; sunfeihu@mail.iee.ac.cn

**Keywords:** heliostat, tracking pose, concentrated solar power, on-line measurement

## Abstract

The tracking pose of heliostats directly affects the stability and working efficiency of concentrated solar power (CSP) plants. Due to occlusion, over-exposure, and uneven illumination caused by mirror reflection, traditional image processing algorithms showed poor performances on the detection and segmentation of heliostats, which impede vision-based 3D measurement of tracking poses of heliostats. To tackle this issue, object detection using deep learning neural networks are exploited. An improved neural network based on YOLO-v5 framework has been designed to solve the on-line detection problem of heliostats. The model achieves a recognition accuracy of 99.7% for the test set, outperforming traditional methods significantly. Based on segmented results, the corner points of each heliostat are found out using Hough Transform and line intersection methods. The 3D poses of each heliostat are then solved out based on the image coordinates of specific feature points and the camera model. Experimental and field test results demonstrate the feasibility of this hybrid approach, which provides a low-cost solution for the monitoring and measurement of tracking poses of the heliostats in CSP.

## 1. Introduction

With the growing demand for renewable energy, concentrated solar power (CSP) has been attracting more and more attention from both industry and academia in recent years. To ensure the concentrated efficiency of a CSP plant, it is essential to monitor and calibrate the tracking attitudes of thousands of heliostats in the field [1]. A large-scale tower-type CSP plant normally has over thousands of heliostats in the field. It is a long, time-consuming process to check the status and orientation of each heliostat using the current beam calibration system.

Beam calibration systems have been traditionally used for the calibration of heliostats in concentrated solar power plants [2,3,4,5,6,7]. Normally, a calibrated high precision CCD imaging system is installed in the field of heliostats. The tracking errors of each heliostat are discovered off-line using a beam calibration system to capture the image of a light spot and compute the deviations of the actual center of the light spot from the aimed position for each heliostat. The beam calibration system gives high accuracy, but the process is too time-consuming, especially for large-size concentrated solar power plants. As heliostats work in hostile outdoor environments, the calibration procedure of heliostats must be carried out every few days. Besides, heliostats cannot work during the calibration process, which directly affects the efficiency of a CSP plant.

Photogrammetric methods that do not interrupt the tracking process of heliostats have been investigated in the past decade. Photogrammetric methods normally involve the task of camera calibration. Given a set of 2D-3D data pairs, different algorithms have been proposed for solving the extrinsic parameters of a camera efficiently [8,9,10,11]. However, extracting the right features from an image and detecting their coordinates at high precision remain a big challenge in many real-world applications. In the case of CSP plants, the field-installed heliostats must rotate around two axes to reflect the sunlight onto the same position on the central receiver from morning to afternoon. The heliostat field images suffer from problems such as over-exposure, uneven illumination, mirror-type reflection, and partial occlusion of objects. Traditional object detection methods [12,13,14,15], such as edge detection, blob detection, template match, and graph theory-based method, face many challenges in heliostat detection due to the existence of these issues.

Digital image analysis techniques have been used to find different types of errors of a small-size single-facet heliostat [16]. Jessen proposed a method of using UAV to estimate the orientation errors of heliostats [17]. The heliostat surface is first constructed using an SFM algorithm, and the images are captured by a UAV-borne camera. The orientations of the heliostats are then estimated based on the reconstructed 3D image of the heliostat. A disadvantage is that the estimated orientation is referred to the camera coordinate frame. It is hard to ensure the estimation accuracy and/or pose stability of a UAV-borne imaging system.

Another idea proposed by researchers is to install four cameras around the central receiver and to estimate the orientation deviation of a heliostat based on the images captured by the four cameras [18,19]. Obviously, this method depends much on the stability of illumination and can only adjust one heliostat at a time, making it complex for realization and very time-consuming.

Several pin-hole cameras were installed at the crevices of the central receiver tubes to monitor the distribution of light flux reflected by the heliostats so that the orientation of the heliostats could be monitored [20]. This method differed from other calibration methods in its direct imaging of luminous flux formed by the heliostats. This method met huge difficulties on its realization due to the high-temperature hostile environment and limited space between the tubes of the receiver. Besides, it is very difficult to discriminate the exact influence of each specific heliostat in the optical flux. Prahl et al. used a series of pictures of a heliostat moving around the azimuth and elevation axes to find the geometric change between the camera and the normal direction of the heliostat to calculate the inclining angle of the heliostat surface [21]. Researchers have also proposed the method of using fixed objects in the environment, such as the star [22], the structures on the concentrated tower, LED lights installed in the field [23], etc., as references to correct the orientation of heliostats. Fairman proposed a method of installing a camera on each heliostat and several artificial targets on the central receiver for automatic calibration of heliostats [22]. The positions of the targets are previously measured using a total station or laser tracker. The poses of each heliostat are then estimated based on the captured image and digital image analysis of targets. This method requires the installation of a camera for each heliostat; the initial hardware cost for a CSP plant, which normally has thousands of heliostats, is unacceptable. In addition, the maintenance expense of these cameras would also be a big burden for the CSP station.

Carballo [24] exploited the application of Faster-RNN on objection detection and a heliostat tracking control system. According to the paper, a Raspberry-Pi-based camera is installed on a camera to capture the target (the white targeting board on the central tower), and the aiming deviation of the heliostat is computed based on target detection and image analysis. The tracking accuracy of the modified heliostat is about 3 mrad during steady-state periods, but it turns worse during transient periods due to the rotating movement of the heliostats. 

## 2. Materials and Methods

**Figure 1:**
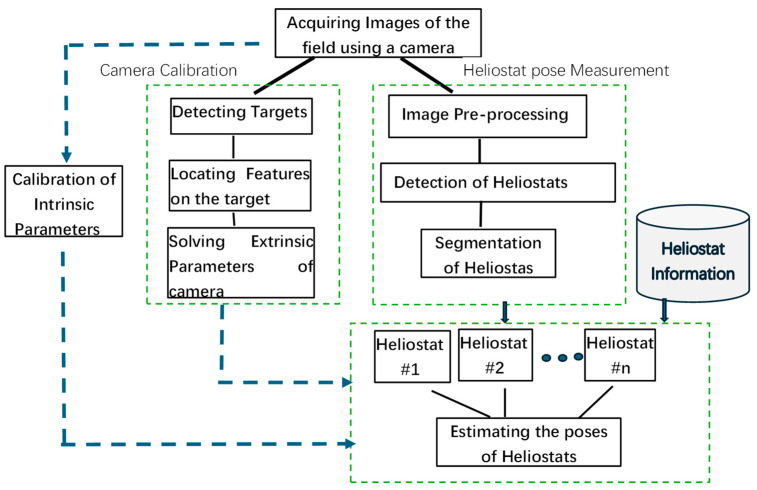
Illustration for 3D pose measurement of heliostats.

In this paper, an approach using a camera installed on the receiver tower and combining a YOLO-based object detection model is proposed for on-line measurement of the tracking poses of heliostats in a CSP plant. The extrinsic parameters of the camera can be calibrated on-line using four fixed targets distributed in the heliostat field. The tracking poses of the heliostats are solved out based on the captured image and digital image analysis technique. The working flow of the 3D pose measuring system for heliostats is shown in Figure 1. Theories and methodology will be introduced in the rest of this section.

### 2.1. On-Line Calibration of Camera

An industry-grade digital camera, made by HIKVISION, Hangzhou, China, is installed on the central receiver tower of the concentrated solar power plant for image acquisition. The camera is equipped with an optical lens of 35 mm focal length. The resolution of the image sensor within the camera is 4096 × 2160. The intrinsic and extrinsic parameters of a camera decide the transform function between the 3D physical point and the corresponding image point. Given a camera with fixed focal length, its intrinsic parameters normally remain constant. The accurate values of intrinsic parameters can thus be calibrated in advance at an indoor laboratory using Zhang’s method [9]. The extrinsic parameters of the camera, however, must be calibrated on-line because of the uncertainty brought by the field installation and possible disturbances.

**Figure 2:**
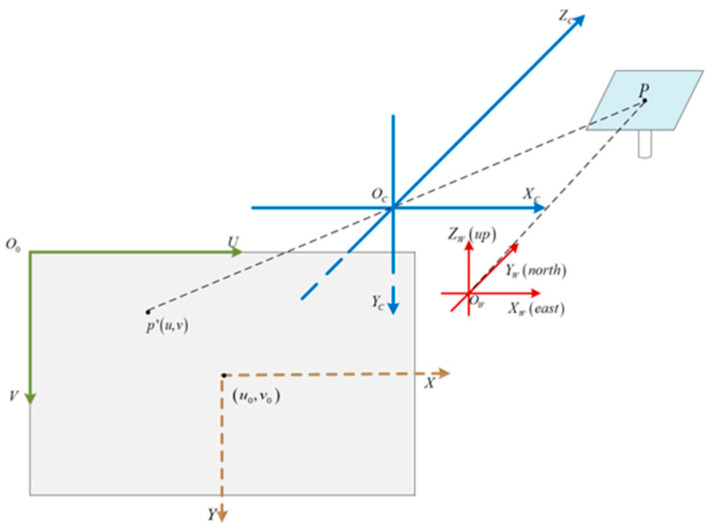
Illustration of a pin-hole imaging system.

The pin-hole model, as shown in Figure 2, is widely used for imaging with a single-lens camera. Supposing the coordinates of point P in the world coordinate frame are indicated as (*X_w_*, *Y_w_*, *Z_w_*), the line connecting point P and the principal point of the camera (the origin of camera frame XcYcZc) intersects with the imaging plane at point p’. The coordinates of p’ in the image coordinate frame are denoted as (u, v) in pixels. The transformation of a physical point P in the world coordinate frame to the image point p’ in the image coordinate frame can be described using Equation (1), as follows:

(1) $$s\left[\begin{array}{c} u\\ v\\ 1 \end{array}\right]=\left[\begin{array}{ccc} f_{x} & 0 & u_{0}\\ 0 & f_{y} & v_{0}\\ 0 & 0 & 1 \end{array}\right]\left[\begin{array}{cc} R & t\\ 0^{T} & 1 \end{array}\right]\left[\begin{array}{c} X_{w}\\ Y_{w}\\ Z_{w}\\ 1 \end{array}\right]={Μ}_{I}{Μ}_{E}\left[\begin{array}{c} X_{w}\\ Y_{w}\\ Z_{w}\\ 1 \end{array}\right]$$

In Equation (1), *s* is a non-zero scaling factor, *f_x_* and *f_y_* represent the normalized focal length along the horizontal and vertical directions of the image sensor, respectively, and *u*_0_ and *v*_0_ are the coordinates of the principal point in the image. Matrices *M_I_* and *M_E_* contain the intrinsic and extrinsic parameters of the imaging system, respectively. Given a set of points with known world coordinates and 2D image coordinates, the intrinsic and extrinsic parameters can be solved out using the direct linear transform (DLT) method or Faugeras calibration method [6]. However, there is a prerequisite for applying these methods; i.e., the 3D world coordinates of the feature points (at least six points) must be known.

When the intrinsic parameters are known, the extrinsic parameters of the camera can be obtained using Efficient Point-n-Perspective (EPnP) algorithm. The main idea of EPnP is introducing virtual control points to describe the physical points in the world coordinate frame so that the computation complexity of solving PnP can be reduced. The principle is introduced in the following equations.

Assuming $p_{i}^{w},p_{i}^{c},\;and\;C_{j}^{w},C_{j}^{C}(i=1,2,\cdots ,n;j=1,2,3,4)$ denote the coordinates of n reference points and four virtual control points in the world and camera coordinate frame, respectively, we have the following equation:

(2) $$p_{i}^{w}=\sum_{j=1}^{4}{\alpha_{ij}C}_{j}^{w}$$

where $\alpha_{ij}$ are co-efficients, satisfying the normalization condition. 

As the rotation matrix between the camera coordinate frame and world coordinates frame is $\left[\begin{array}{cc} R & t \end{array}\right]$, there are:

(3) $$\left.p_{i}^{c}=\left[\begin{array}{cc} R & t \end{array}\right.\right]\left[\begin{array}{c} p_{i}^{w}\\ 1 \end{array}\right]$$

(4) $$\left.C_{j}^{C}=\left[\begin{array}{cc} R & t \end{array}\right.\right]\left[\begin{array}{c} C_{j}^{W}\\ 1 \end{array}\right]$$

By combining Equations (2) and (3), we have:

(5) $$\begin{array}{c} p_{i}^{c}=\left[\begin{array}{cc} R & t \end{array}\right]\left[\begin{array}{c} \sum_{j=1}^{4}\;\alpha_{ij}C_{j}^{W}\\ 1 \end{array}\right]=\sum_{j=1}^{4}\;\alpha_{ij}\left[\begin{array}{cc} R & t \end{array}\right]\left[\begin{array}{c} C_{j}^{W}\\ 1 \end{array}\right]=\sum_{j=1}^{4}\;\alpha_{ij}C_{j}^{C} \end{array}$$

According to the pin-hole imaging model, the image coordinates of the reference points are equal to the product of the intrinsic matrix with the coordinates of the point in the camera frame. Combining with Equation (5), we have Equation (6):

(6) $$\lambda_{i}\left[\begin{array}{c} u_{i}\\ v_{i}\\ 1 \end{array}\right]={Μ}_{I}\sum_{j=1}^{4}\alpha_{ij}C_{j}^{C}$$

Supposing the coordinates of the four virtual control points are denoted as $\left(x_{j}^{C},y_{j}^{C},z_{j\;}^{C}\right)$, j = 1, 2, 3, 4, in the camera frame, Equation (6) can be replaced by the following two equations:

(7) $$\sum_{j=1}^{4}\;\alpha_{ij}f_{u}x_{j}^{C}+\sum_{j=1}^{4}\;\alpha_{ij}\left(u_{0}-u_{i}\right)z_{j}^{C}=0$$

(8) $$\sum_{j=1}^{4}\;\alpha_{ij}f_{v}y_{j}^{C}+\sum_{j=1}^{4}\;\alpha_{ij}\left(v_{0}-v_{i}\right)z_{j}^{C}=0$$

The coordinates of four control points $C_{j}^{C}=\left(x_{j}^{C},y_{j}^{C},z_{j}^{C}\right)$ in the camera coordinate frame are unknown values in Equation (8). Given n pairs of points, we will have 2n equations:

(9) $$A_{2n\times 12}X_{12\times 1}=0$$

Solving the linear system of Equation (9), we can obtain the values of X; i.e., the coordinates of four control points in the camera frame. X can be further expressed, as in Equation (10):

(10) $$X=\sum_{k=1}^{N}\beta_{k}v_{k}$$

where $v_{k}$ are the columns of the right-singular vectors of matrix A and can be found as the null eigenvectors of A^T^A. To find the most appropriate singular values, solutions for all possible value N $\in \left\{1,\;2,\;3,\;4\right\}$ are evaluated. The one with the smallest re-projection error of all reference points is considered as the right value. The value of $\beta_{k}$ is found out based on the constraint that the distances between two control points remain the same in the world and camera coordinate frame, as illustrated in Equation (11):

(11) $$\mathrm{Min}\text{ \{}\mathrm{Error}\left(\beta \right)=\underset{(i,j)s.t.i<j}{\sum }{\left({\left\|\sum_{k=1}^{N}\beta_{k}v_{k}^{i}-\sum_{k=1}^{N}\beta_{k}v_{k}^{j}\right\|}^{2}-{\left\|C_{i}^{W}-C_{j}^{W}\right\|}^{2}\right)}^{2}\}$$

Here, $i,j\in \left[1,4\right]$, are the indexes of the control points.

After the camera is installed on the tower, the extrinsic parameters of the camera can thus be calibrated based on captured images of the heliostat field. The calibration procedures go through the following steps:

(1) Select images from the repository of captured images of the heliostat field;
(2) Detect the targets and locate the feature points on the image;
(3) Obtain the coordinates of feature points based on the captured time and known information of the target;
(4) Initialize the rotation matrix of the camera;
(5) Use the bundle adjustment method to find the optimal extrinsic parameters of the camera based on the minimization of re-projection errors of selected feature points.

### 2.2. Detection of Heliostats

Object detection and segmentation are fundamental tasks of vision-based applications. For most industrial indoor vision-based applications, traditional image processing algorithms can fulfill the requirements withs proper design of illumination and optical imaging system. For outdoor applications, the task of object detection and segmentation becomes challenging due to the disturbances of the environment.

#### 2.2.1. Object Detection Model

Object detection based on deep learning models has been widely investigated in the last decade. Several deep neural network models, such as R-CNN, Fast R-CNN, YOLO, SSD, etc. [25,26,27], have been designed and developed for object detection.

**Figure 3:**
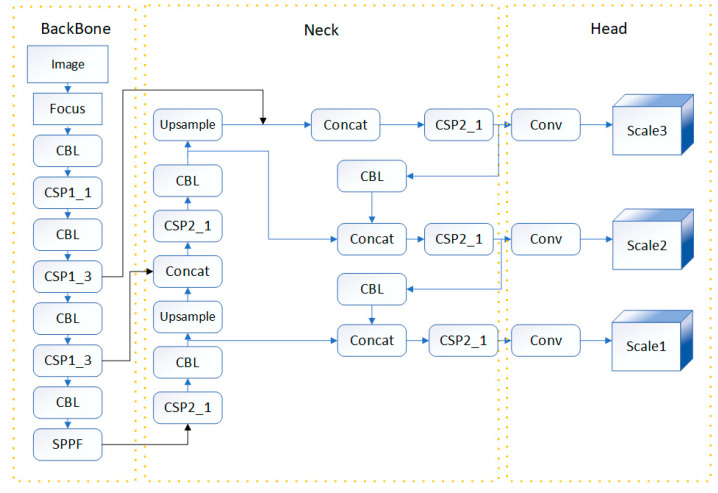
The network structure of YOLOv5.

Among them, the YOLO (You Only Look Once) model has achieved big success due to its one-stage structure and ability to handle multi-scale object information. The YOLOv5 model consists of three parts: the backbone for feature extraction, the neck for feature fusion, and the head for object detection, as illustrated in Figure 3.

At the backbone part, the image goes through the Focus layer, a combination of CBL (Convolution, Batch normalization, Leaky Relu) operations, Cross Stage Partial (CSP) modules, and SPPF (Spatial Pyramid Pooling with Fusion) module to extract rich features of different scales.

The feature maps are fed to the neck part for feature fusion. The network of the neck part adopts both Feature Pyramid Network (FPN) structure and Path Aggregation Network (PAN) structure [28,29]. The FPN structure [30] is responsible for transferring strong semantic features from top-level feature maps to bottom-level feature maps, while the PAN structure [31] transfers stronger localization features from bottom-level feature maps to top-level feature maps. The collaborative effect of these two structures significantly enhances the feature fusion capability of the neck network.

YOLOv5 adopts Generalized Intersection over Union (GIoU) as the loss function for bounding box regression. Let A be the predicted box, B be the ground truth box, and C be the smallest convex hull containing both A and B. The calculation formula for GIoU is given in Equation (12), and the computation of the GIoU loss function is described in Equation (13).

(12) $$GIoU=IoU-\frac{\left|A_{c}-U\right|}{\left|A_{c}\right|}$$

(13) GIoULoss =1− GIoU

YOLOv5 has achieved a balance between speed and accuracy, making it suitable for real-time object detection. However, the detection accuracy with the YOLOv5 model is not very satisfactory for images with regional over-exposure. Regional over-exposure occurs often in the captured image due to the movement of heliostats and deviated reflections from the heliostat surfaces. Therefore, an attention-enhanced YOLO model is proposed. 

#### 2.2.2. Attention-Enhanced Object Detection

Attention mechanism can extract more crucial information by putting greater weights on the important regions of an input image. In practice, there are various implementations of attention mechanisms [31,32,33]. A simple, parameter-free attention module (SimAM) has been proposed by Yang et al. recently [34]. Different from existing attention modules, SimAM generates three-dimensional attention weights without introducing additional parameters to the original network, therefore called parameter-free.

SimAM utilizes an energy function, as shown in Equation (14), to compute attention weights for each neuron in the network:

(14) $$e_{t}\left(w_{t},b_{t},y,x_{i}\right)=\frac{1}{M-1}\sum_{i=1}^{M-1}\;{\left(-1-\left(w_{t}x_{i}+b_{t}\right)\right)}^{2}+{\left(1-\left(w_{t}t+b_{t}\right)\right)}^{2}+\lambda w_{t}^{2}\;$$

In Equation (14), t and $x_{i}$ are the target neuron and other neurons in a single channel of the input feature $X\in R^{C\times H\times W}$, respectively; i is an index, M=H×W is the number of neurons on that channel, H is the height of the image, W is the width of the image, and $w_{t}$ and $b_{t}$ are the weight and bias for transformation. 

The minimization of function (14) can lead to an analytical solution; thus, the minimum energy can be obtained through the following expression:

(15) $$\;e_{t}^{*}=\frac{4\left(\hat{\sigma^{2}}+\lambda \right)}{{\left(t-\hat{u}\right)}^{2}+2{\hat{\sigma }}^{2}+2\lambda }$$

where $\hat{u}=\frac{1}{M-1}\sum_{i=1}^{M-1}x_{i},{\hat{\sigma }}^{2}=\frac{1}{M-1}\sum_{i=1}^{M-1}{\left(x_{i}-\hat{u}\right)}^{2}$.

The above formula indicates that the lower the energy, the greater the contrast between the neuron t and the surrounding neurons, indicating higher importance. Therefore, the importance of neurons can be obtained as the reciprocal of $e_{t}^{*}$.

Specifically, enhancement processing is applied to the features as described in Equation (16):

(16) $$\tilde{X}=sigmoid\left(\frac{1}{E}\right)⊙X$$

where E groups all $e_{t}^{*}$ across channel and spatial dimensions, and the addition of the Sigmoid function is introduced to limit the large values of E without affecting the relative importance of each neuron.

**Figure 4:**
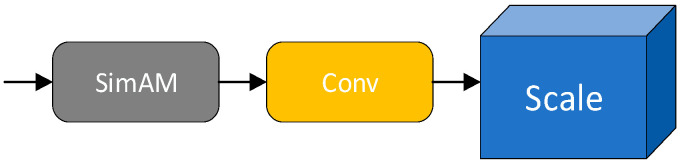
Addition of SimAM attention modules.

To optimize the model’s performance, a SimAM attention module is added before each Convolution module in the Head network of YOLOv5, as depicted in Figure 4. This enhancement reinforces the network’s focus from low-level texture features to high-level semantic features. A model using the SimAM-enhanced YOLO network has been trained for heliostat detection. Experiments show that the addition of the SimAM module has improved the object detection accuracy in this application.

### 2.3. Segmentation of Heliostats

ROI (Region of Interest) represents the region of interest in an image, typically containing the object being detected [35,36]. The detected ROIs returned by the YOLOv5s-SimAM model must be post-processed to obtain the coordinates of the upper left corner of the object as well as the length and width of the enclosing box. These data are crucial for locating the position and size of each heliostat in the image. Using ROI to divide the image into a set of sub images containing the heliostat, image processing procedures are then carried out on these sub images to find out the coordinates of corner points of each heliostat.

#### 2.3.1. Noise Reduction and Binarization

Due to high reflectivity of the mirror surfaces of heliostats, ground as well as other objects in the field of view of the camera are normally very dark in the captured image. This makes post-processing of ROI relatively easier.

To reduce the influence of noise, gaussian filtering is applied at first. Compared with mean and median filtering, gaussian filtering weights the pixels within the template. The template has a characteristic of circular symmetry, thereby better preserving the detailed structural information within the image.

Global thresholding is used for the binarization of the image. The threshold is determined based on the median intensity value of the image. After binarization, morphological operations are applied to fill the small holes and gaps in the cropped image.

#### 2.3.2. Determination of Corner Points

Canny edge detector is applied to find the edges in the cropped image. The canny edge detector includes the following steps:

(1) The magnitude of the gradient $G_{xy}(i,j)$ and the direction θ(i,j) are calculated using the following formulas:
  (17) $$G_{xy}(i,j)=\sqrt{G_{x}(i,j)+G_{y}(i,j)}$$
  (18) $$\theta (i,j)=artan\left(\frac{G_{y}(i,j)}{G_{x}(i,j)}\right)$$
  where x and y represent the horizontal and vertical direction, and i and j are pixel index.
(2) Based on the gradient magnitude and direction, all pixels are traversed to determine whether their gradient magnitude is a local maximum in the direction of their gradient. If it is, the pixel is retained; otherwise, it is set to 0.
(3) Two thresholds, t_min_ and t_max_, are set. Pixels with gradient magnitudes greater than t_max_ are classified as edge pixels and retained; pixels with gradient magnitudes less than t_min_ are considered non-edge pixels and discarded. For pixels with gradient magnitudes between t_min_ and t_max_, if they are connected to pixels classified as edge pixels by the algorithm, they are considered part of the edge; otherwise, they are discarded.

After canny operation, Hough Transform (HT) is used for the detection of boundaries of the heliostat in each cropped image. Using Hough Transform, a line in the x-y plane can be denoted as a point in the Hough parameter space, as illustrated in Equation (19).

(19) ρ=xcosθ+ysinθ

In Equation (19), ρ is the distance from the line to the origin, and θ represents the angle between the line and the x axis. The range of θ is typically from 0 to π, indicating the angle measured counterclockwise from the positive x axis. In practice, the parameters θ and ρ are quantized to obtain an array C. The value of each cell in array C is decided on the hits of edge pixels in the image. Each local maxima in C then represents a possible line segment in the image.

As the heliostat has a rectangular shape, the external boundaries and inner boundary of sub facets are all linear segments in the image. Four peak points in the Hough parameter space are identified as the four external boundary edges of the heliostat. Finding the parameters of four boundary edges, the four corner points can be determined by calculating their intersections.

### 2.4. Calculating the Heliostat Pose

Given the imaging model of the camera, the tracking pose of the heliostat in the world coordinate frame can be solved out based on the image coordinates of the feature points and the geometrical constraints of the heliostat.

**Figure 5:**
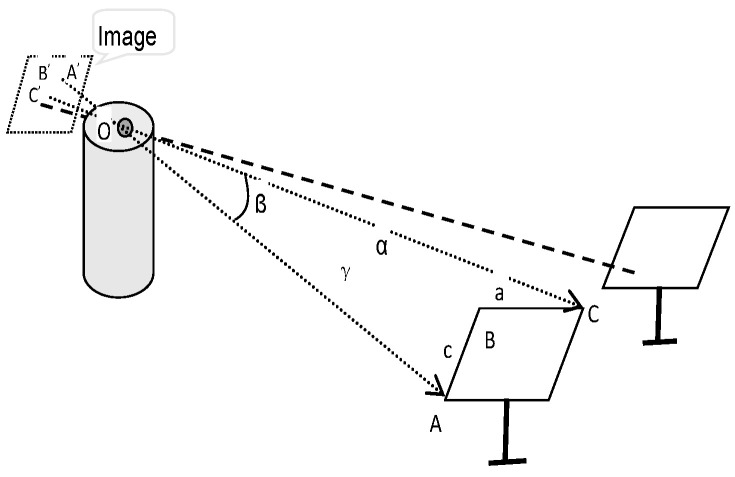
Illustration of projection of heliostat in the image.

Considering a heliostat in the field, the corner points of the heliostat are feature points and are denoted as A′, B′, and C′ in the image plane, as illustrated in Figure 5. As the mirror surface of the heliostat is a rigid structure of rectangular shape, its width and height are fixed values. In Figure 5, the width of the heliostat is denoted as *a*, the height of the heliostat is denoted as *c*, the straight distance between point C and A is denoted as *b*, the principal point of the camera is denoted as O, the distance between points A and O is denoted as *d*_1_, the distance between points B and O is denoted as *d*_2_, and the distance between points C and O is denoted as *d*_3_.

The angle between the line of OB and OC is denoted as α, the angle between OA and OC is β, and the intersecting angle of OA and OB is γ. These angles can be computed using the dot product of corresponding unit directional vectors in the camera frame, as shown in Equation (20):

(20) $$\left\{\begin{array}{c} cos\alpha =e_{2}^{T}e_{3}\\ cos\beta =e_{1}^{T}e_{3}\\ cos\gamma =e_{1}^{T}e_{2} \end{array}\right.$$

In Equation (20), *e*_1_ is the unit directional vector of OA, which can be estimated based on the image coordinates of *A*′ on the normalized imaging plane, namely, [*x_AC_*, *y_AC_*, 1], as shown in Equation (21). The vector [*x_AC_*, *y_AC_*, 1] can be obtained by multiplying the pixel coordinates of the corresponding point in the image with the inverse of the intrinsic matrix of the camera. Similarly, *e*_2_ is the unit directional vector of line OB, and *e*_3_ is the unit directional vector of line OC. They can be computed in the same way, as follows:

(21) $$e_{1}=\frac{1}{\sqrt{{x^{2}}_{AC}+y_{AC}^{2}+1}}\left[\begin{array}{c} x_{AC}\\ y_{AC}\\ 1 \end{array}\right]$$

Based on the affine geometry, the following equations are established:

(22) $$d_{2}^{2}+d_{3}^{2}-2d_{2}d_{3}cos\alpha =a^{2}\;d_{1}^{2}+d_{3}^{2}-2d_{1}d_{3}cos\beta =b^{2}\;d_{1}^{2}+d_{2}^{2}-2d_{1}d_{2}cos\gamma =c^{2}$$

Solve the equations to find out *d*_1_, *d*_2_, and *d*_3_. The coordinates of corner points A, B, and C in the camera frame can be calculated based on the following equation:

(23) $$\vec{OA}=d_{1}\vec{e_{1}}\;\vec{OB}=d_{2}\vec{e_{2}}\;\vec{OC}=d_{3}\vec{e_{3}}$$

With three points, we can acquire the rotation matrix ^m^Rc of the heliostat surface in the camera coordinate frame. As the rotation matrix between the camera coordinate frame and the world coordinate frame is known, we can obtain the rotation matrix of the heliostat surface with respect to the world coordinate frame, i.e., Rwm:

(24) Rwm=RcmRwc

The rotation matrix of heliostat Rwm is decided by the three rotating angles of the heliostat, namely

(25) $$R\left(\alpha ,\beta ,\gamma \right)=R_{z}\left(\alpha \right)R_{x}\left(\beta \right)R_{y}\left(\gamma \right)\;=\left[\begin{array}{ccc} cos\alpha cos\gamma -cos\beta sin\alpha sin\gamma & -cos\beta cos\gamma sin\alpha -cos\alpha sin\gamma & sin\alpha sin\beta \\ cos\gamma sin\alpha +cos\alpha cos\beta sin\gamma & cos\alpha cos\beta cos\gamma -sin\alpha sin\gamma & -cos\alpha sin\beta \\ sin\beta sin\gamma & cos\gamma sin\beta & cos\beta \end{array}\right]$$

where $R_{z}\left(\alpha \right)$ indicates the rotating angle around the z axis, $R_{x}\left(\beta \right)$ indicates the rotating angle around x axis, and $R_{y}\left(\gamma \right)\;$ is the rotating angle around the y axis. For azimuth-inclination two-axis driven heliostat, the rotating angle $\;R_{y}\left(\gamma \right)$ is normally near zero.

The azimuth angle *yaw* and the inclination angle *pitch* can be calculated based on Equation (26), as follows:

(26) $$yaw=atan2(R_{21},R_{11})\;pitch=atan2\left(-R_{31},\sqrt{{R_{32}}^{2}+{R_{33}}^{2}}\right)$$

where $R_{ij}$ indicates the element located at the *ith* row and *jth* column of the rotation matrix Rwm.

## 3. Results

**Figure 6:**
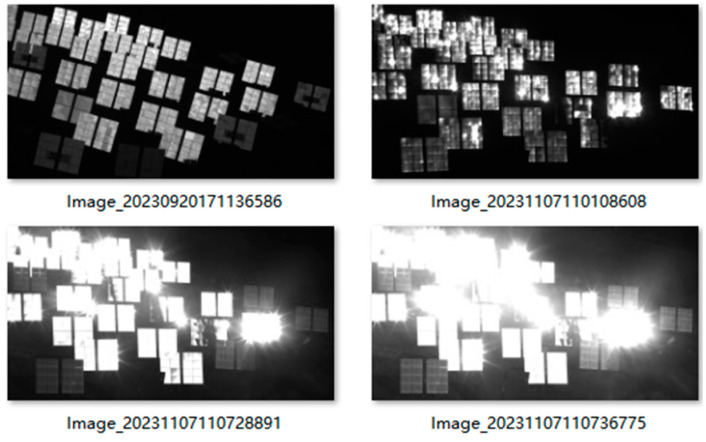
Representative samples in the dataset.

Images of the heliostat field under different lighting conditions and with various heliostat postures are captured. In the data collection process, a total of 341 images of the heliostat field were gathered. All images take the same size of 4096 × 2160, which is the resolution of the camera. Figure 6 shows four samples of the image dataset of heliostat fields. To enrich the number and diversity of samples, mosaic data augmentation has been applied to the dataset. The core idea of mosaic data augmentation is to select four images from the dataset at random and stitch them into a new composite image. The constructed dataset of the heliostat field is split into three sets: the training set, the test set, and the validation set, in a ratio of 6:1:1.

**Figure 7:**
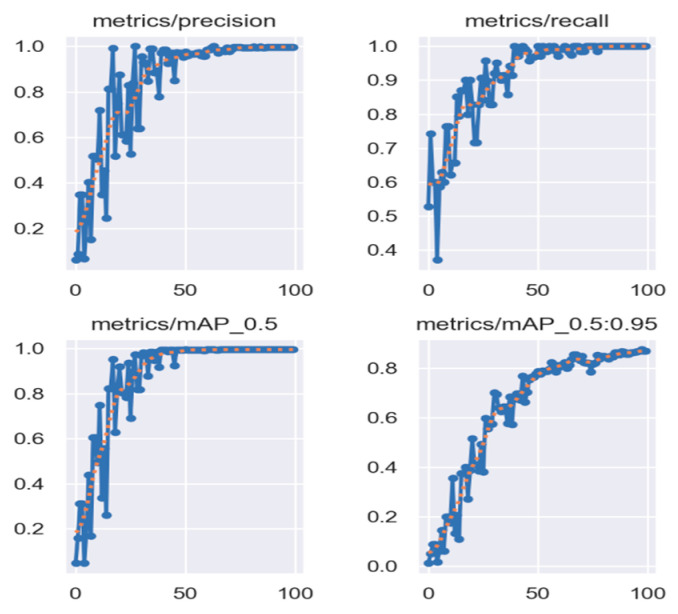
Training process.

The training process of the YOLOv5s-SimAM model is shown in Figure 7. The iteration converges around the 50th epoch, with a very promising result.

**Figure 8:**
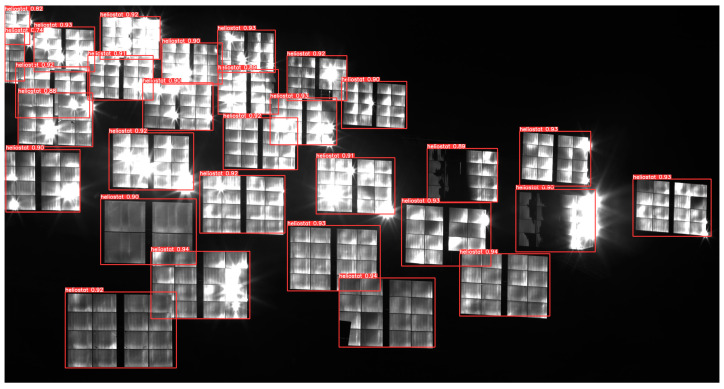
Image of heliostat recognition.

An example of heliostat recognition result using the YOLOv5s-SimAM model is shown in Figure 8. Almost all heliostat objects, even the heliostats with partial occlusion, have been successfully detected in the example. The precision, recall, and mAP (mean average precision) of the proposed YOLOv5s-SimAM are much better than that of the original YOLOv5s model and slightly better than that of the YOLOv8s model, as shown in Table 1.

Table 2 gives a comparison of the performances of traditional methods. Twenty images of the heliostat field, each containing 28 heliostats, were selected for test and comparison. In total, there are 560 instances of heliostats in the test images. Due to over-exposure and partial occlusion, traditional object detection methods, such as templated matching, contour detection, and K-means clustering-based method, failed to detect many of the heliostat objects in the test images. In contrast, the YOLO model detected most of the objects in the test images. The proposed YOLOv5s-SimAM achieved a recognition accuracy of 99.4% on the test images.

### 3.1. Segmentation Result

**Figure 9:**
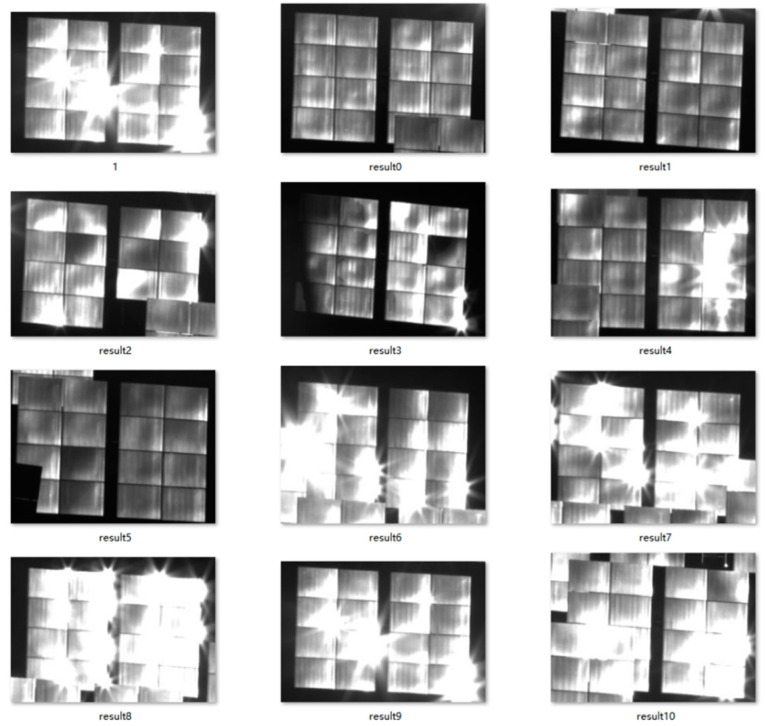
Detected objects of the heliostat.

After detecting the multiple objects of the heliostat, each heliostat is cropped along the enclosing rectangle of detected ROIs. The segmentation results obtained using this method, particularly for heliostat images captured at noon time, are displayed in Figure 9. One can see that the boundaries of the heliostat are not clear in many detected ROIs.

**Figure 10:**
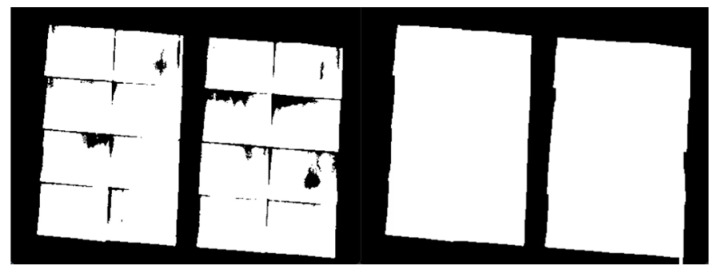
Binary image (**left**) and processed binary image (**right**).

**Figure 11:**
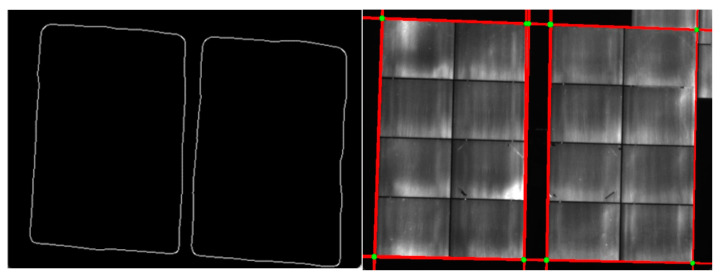
Detected contour (**left**) and detected corner points (green points in the (**right image**)).

As the mirror surface of heliostat consists of 16 rectangular facets, there are many line segments in the processed image. Hough Transform is used to find the external boundary of the heliostat surface. The corner points are then found out through the intersection of edges, as introduced in Section 2.3. An example of the processed heliostats is shown in Figure 10 and Figure 11.

### 3.2. Measurement Result

The goal of this project is to measure the tracking poses of multiple heliostats without interfering their normal sun-tracking procedures. Based on the method introduced in Section 3, an application software has been developed in C++. Experiments for on-line measuring of the tracking poses of heliostats have been arranged and carried out in a concentrated solar plant located in Hebei Province. The tracking poses of five heliostats measured with the method proposed in this paper are recorded and compared to the actual tracking angles, as shown in Table 3. The actual tracking angles are given by the beam calibration system, which was installed in the field of CSP previously for the purpose of heliostat calibration.

**Figure 12:**
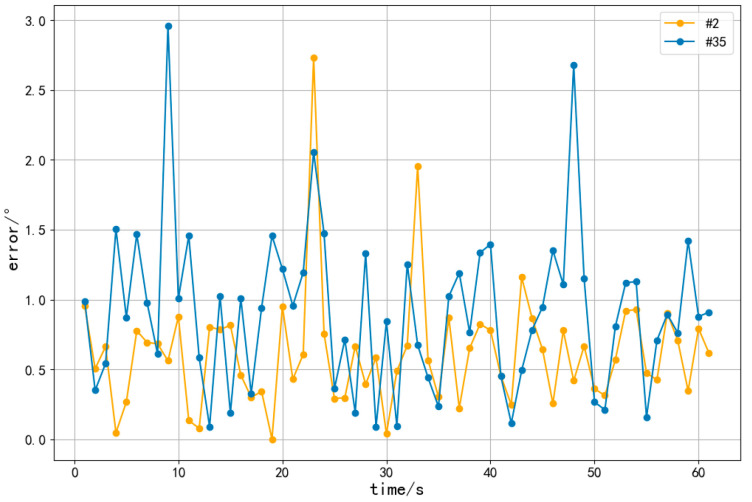
Errors of the azimuth angle within one minute.

**Figure 13:**
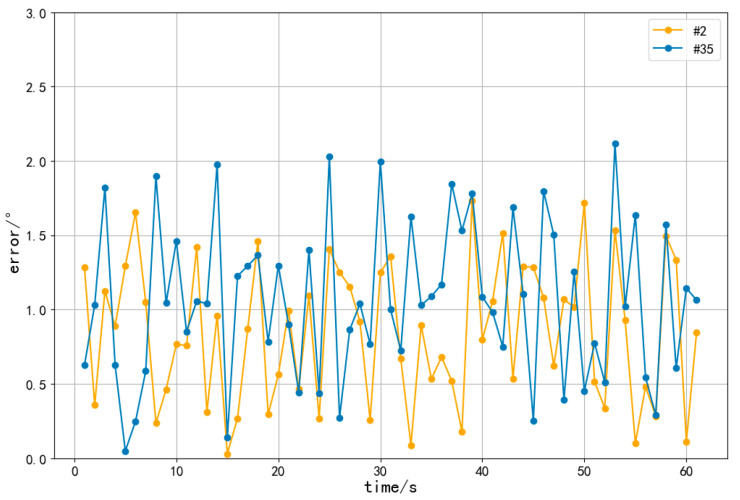
Errors of the inclination angle within one minute.

To analyze the measuring characteristics of the proposed method, experimental results of two heliostats, the #2 and #35, were collected within one minute. The calculation was performed every one second. The errors of azimuth and inclination angles of these two heliostats were calculated and plotted, as shown in Figure 12 and Figure 13. 

The mean error of azimuth angle for the #2 heliostat within one minute is 0.62°, and it is 0.91° for the #35 heliostat. The mean error of the inclination angle for the #2 solar mirror within one minute is 0.85°, and it is 1.06° for the #35 heliostat.

The errors of both the azimuth and inclination angles for the #35 heliostat are greater than those of the #2 heliostat. This is due to the greater distance between the #35 heliostat and the central tower. The line-of-sight distance of the #2 heliostat from the camera is about 94 m, while the #35 heliostat is located about 128 m away from the camera.

## 4. Conclusions

The operation efficiency of concentrated solar power plants is directly affected by the tracking accuracy of the heliostats. The traditional beam calibration system uses a Lambert surface as the aiming target and calculates the tracking error based on the deviation of a concentrated solar spot. Such methods can only calibrate heliostats one by one in an off-line mode, making the calibration process a time-consuming task. This paper proposes an approach for on-line monitoring and measurement of the tracking poses of heliostats in concentrated solar power plants. The proposed approach can calculate the tracking poses of all heliostats within the field of view of the camera at the same time and without disturbing the normal working procedure of each heliostat.

The measurement accuracy of the proposed approach relies on the detection accuracy and segmentation precision of heliostats. Due to issues such as uneven illumination caused by mirror reflection and mutual occlusion of heliostats, traditional image processing algorithms often failed to detect and segment the objects of heliostats in the image. To solve this problem, a deep learning model based on YOLOv5 framework and attention module has been trained for on-line detection of heliostats. The recognition accuracy of the model on the collected dataset reached 99.7%, meeting the requirement of the on-line detection task. Meanwhile, traditional image processing methods are used for segmentation of heliostats and for detection of the four corner points of the heliostat. Based on the detected 2D image coordinates of four corner points, the rotation matrix of the heliostat coordinate frame relative to the camera coordinate frame can be obtained. As the rotation matrix of the installed camera can be calibrated in advance, the rotation matrix of the heliostats in the world coordinate frame can be determined. The tracking poses of heliostats during the sun-tracking process can thus be solved based on the captured images of the heliostat field.

The proposed approach has been tested in a concentrated solar power plant, using a PC from Lenovo. The PC is equipped with an Intel i7-12700 processor and a Nvidia RTX 3060 GPU board (Lenovo, Beijing, China). The time for measuring the tracking poses of all 28 heliostats within the field of view of the camera costs about 1.5 s. Considering the relative movement of the sun is about 0.004 degrees per second, such speed is acceptable for on-line monitoring of the tracking poses of heliostats. The measuring time can be further reduced by improving the software and using a faster computer.

## Figures and Tables

**Table 1:** Comparison of various YOLO models.

Model	Precision	Recall	mAP	mAP 0.5:0.95
YOLOv8s	0.986	0.998	0.995	0.940
YOLOv5s	0.972	0.995	0.993	0.762
YOLOv5s-SE	0.985	0.998	0.994	0.791
YOLOv5s-SimAM	0.997	1.000	0.995	0.868

**Table 2:** Comparison with traditional methods.

Methods	Correctly Identified	Precision
Template matching	89	0.159
Contour detection	243	0.434
K-Means	76	0.136
YOLOv8	552	0.986
YOLOv5s-SimAM	557	0.994

**Table 3:** Measured poses of five heliostats.

#No. of Helio.	Dist. (Meter)	Azimu. (Actual)	Azimu. (Meas.)	Inclin. (Actual)	Inclin. (Meas.)
#2	94	128.81	129.28	66.52	67.29
#17	110	133.88	135.66	61.67	60.95
#36	127	141.04	140.74	72.71	71.24
#44	135	136.65	138.23	72.18	76.10
#47	136	126.31	124.92	71.28	65.54

## Data Availability

Data are contained within the article.
